# 15. Uncommon CNS manifestation in systemic lupus erythematosus

**DOI:** 10.1093/rap/rky033.007

**Published:** 2018-09-20

**Authors:** May Zun Swe, Ameen Jubber, Rachel Jeffery

**Affiliations:** Rheumatology, Northampton General Hospital, Northampton, UNITED KINGDOM

**Introduction:** Systemic lupus erythematosus (SLE) can be associated with various neuropsychiatric manifestations affecting the nervous system at multiple levels, with differing neuropathology. Among them, transverse myelitis is one of the rare neurologic syndromes that can present with the rapid onset of weakness, sensory alterations and bowel, bladder or sexual dysfunction. This syndrome is thought to be due to an arteritis, with resultant ischemic necrosis of the spinal cord, which has been associated with anti phospholipid antibodies (aPL) in some studies.

**Case description:** A 30 year old female with quiescent SLE on hydroxychloroquine 200 mg/400 mg alternate days, had been diagnosed in 2010 with features characterised by fatigue, arthralgias, a short-lived malar rash, resolved low level proteinuria, ANA and anti-Ro positivity. She presented in 2015 with an eight-week fluctuating history of sudden onset significant pain in her neck, which radiated into her shoulders bilaterally associated with some tingling down the medial aspect of both arms into the medial two digits. She also reported some decline in dexterity in both hands with bilateral weak finger grip worse on the left side as well as frequent headaches. However, there was no symptomatic evidence of sphincter or lower limb dysfunction at that time. Cranial nerve examination including fundoscopy was normal. On assessment of co-ordination, however, she had as a subtle action and postural tremor of left arm. In addition, there was mild weakness distally in the hands worse on the left side, particularly in finger extension and finger abduction. Of note, her reflexes were present and symmetrical. MRI imaging of the head was normal. MRI of the spine and subsequent gadolinium enhanced imaging demonstrated an area of expansion of the upper thoracic cord from T1 to T3, with features of generalised oedema of the cord and intramedullary signal change between C7 and T3. Specialist neuroradiological review suggested that this was more in keeping with transverse myelitis than a neoplasm. What is more, lumbar puncture showed raised CSF protein (0.83g/L), no organisms on culture or PCR and no malignant cells. She was treated with oral methylprednisolone 250 mg twice daily for five days with bone and gastric protection followed by daily prednisolone reducing dose. She received high dose IV cyclophosphamide (15 mg/kg) for as she continued to have progressive neurological symptoms with lower limb weakness. Following completion of six pulses of IV cyclophosphamide, she was maintained on azathioprine until the end of 2017. Serial MRI imaging of her thoracic and lumbar spine showed no evidence of further progression fortunately. Overtime with neurorehabilitation, this patient has made a good recovery and has not had any further SLE activity. She has had corticosteroid induced side-effects and became very cushingoid, which has improved off treatment. She continues just on hydroxychloroquine treatment.

**Discussion:** Although clinical presentation and MRI imaging features are most suggestive of a transverse myelitis, alternative possibilities such as a focal syrinx, space occupying lesion and multiple sclerosis were considered and ultimately excluded on the serial imaging and clinical progress. Biopsy was not considered a possible or safe option. Although usually a late manifestation of SLE, transverse myelitis can occur at presentation. Interestingly, a longitudinally extensive transverse myelitis has been reported as a phenomenon purely related to underlying SLE but is most commonly seen in neuromyelitis optica or NMO- spectrum disorders which occur in higher frequency in patients who also suffer from SLE. Therefore, it is helpful to perform gadolinium-enhanced MRI spine and antibody profile for neuromyelitis optica spectrum disorders. A sensory level with spastic lower limb weakness and sphincter disturbance is usually the most common presentation, but a thoracic or cervical sensory level can be affected in some patients. Aggressive early treatment is associated with better recovery. In with anti-phospholipid antibodies (which this patient did not have), anticoagulation combined with glucocorticoids and immunosuppressive treatment may achieve an improved outcome.

**Key Learning Points:** Transverse myelitis is a rare complication of systemic lupus erythematosus. Transverse myelitis can occur even when other parameters of SLE are quiescent. On neuroimaging alone, it can be difficult to distinguish causes of spinal cord inflammation and treatment decisions may need to be made on the basis of most likely diagnosis from imaging, history and risk factors and monitored over time.

**Disclosure: M. Swe:** None. **A. Jubber:** None. **R. Jeffery:** None.

